# Preparation and Characterization of Black Seed Oil Oleogel and Its Application in Reduced Animal Fat Beef Hamburger Formulations

**DOI:** 10.1002/fsn3.72257

**Published:** 2026-08-17

**Authors:** Shadieh Roon, Hadi Almasi, Fariba Zeynali

**Affiliations:** ^1^ Department of Food Science and Technology, Faculty of Agriculture Urmia University Urmia Iran

**Keywords:** beeswax, black seed oil, cooking loss, hamburger, oleogel

## Abstract

This study aimed to produce an oleogel with comparable properties to animal fat for using as fat replacer in beef hamburger. Various ratios of beeswax (B) and adipic acid (A) gelators in black seed oil oleogels were investigated at a total concentration of 5% w/w of oil. The combined use of beeswax and adipic acid gelators did not result in any synergistic effects on the textural and rheological characteristics. Sample B5 (beeswax gelator) was selected as the most suitable oleogel. A needle‐shaped crystal morphology and the highest melting point and storage modulus were observed for B5 oleogel. The effects of replacement ratio (6%–12%) and storage time (1, 15, and 30 days) on the physicochemical properties of the hamburgers were evaluated. Increasing the percentage of oleogel replacement led to a decrease in oil absorption (0.45% ± 0.06%), wrinkling (5.14% ± 0.03%), and cooking loss (9.43% ± 0.03%), while the amount of moisture retention increased. However, these parameters showed an increasing trend over time. The oleogel formulated hamburgers had lower TBARS during storage. Overall, this research demonstrated that substituting animal fat with black seed oil oleogel is able to decrease saturated and trans‐fatty acids in beef hamburger.

## Graphical abstract

Oleogel was formulated by Black Seed Oil and beeswax/adipic acid as oleogelator. Beeswax was better to improve the rheological and textural properties of oleogels and was chosen for next step. Beef Hamburger with 6%, 9% and 12% animal fat replacement by oleogel were made and physical properties of oleogel formulated hamburger were studied. Oxidative stability of hamburgers improved by application of oleogel and the physical properties of oleogel‐loaded hamburgers were better than control samples.

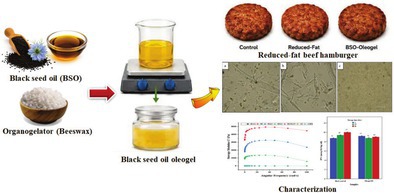

## 1 Introduction

Beef hamburgers, made from processed beef, are one of the most popular processed meat due to their high content of essential nutrients such as proteins, vitamins and minerals (do Prado et al. 2019). However, the nutritional quality of beef hamburgers has been called into question by health experts due to the presence of high levels of saturated fatty acids (SFA) contained in animal fat, which can constitute up to 30% of the product (Heck et al. 2017). As a result, researchers have looked into various options for producing healthier products, including the replacement of saturated fats with unsaturated oils in meat products (Alejandre et al. 2019). Fat replacement in food products is a challenging task due to the significant impact of fat on the textural and sensory properties of food (Kouzounis et al. 2017). Replacing solid animal fat with liquid oils from vegetable or marine sources presents a technical challenge as the function and texture of the fat phase in meat products greatly affects several product properties. To overcome this issue, the conversion of liquid lipids into structured soft matter systems exhibiting functional properties comparable to those of solid fat has been proposed (Gómez‐Estaca, Herrero, et al. 2019; Gómez‐Estaca, Pintado, et al. 2019). One possible approach is to use edible organogels or oleogels, which have garnered recent interest in various fields such as cosmetic, pharmaceutical, and food sciences (Cerqueira et al. 2017). However, in the context of meat products, the use of oleogels as a substitute for animal fat with vegetable oils made by organogelators is a new concept, with limited research focus on minced meat products. Oleogels are thermally reversible viscoelastic materials that encapsulate an organic liquid within a three‐dimensional gel network. In other words, they turn liquid oil into a gel with viscoelastic properties (Jimenez‐Colmenero et al. 2015). Organogels are an attractive alternative for use in food products, owing to their capacity to provide desirable characteristics such as compatibility, flexibility, absence of trans fatty acids (TFA), and a significant reduction in saturated fatty acid (SFA) content (Chaves et al. 2018). Previous studies on oleogels have primarily focused on single‐component oleogels, which have been found to be unable to create structures similar to common fats. Consequently, researchers have shifted their attention towards investigating combined oleogels and the interaction between different gelators. Hybrid oleogels have superior stability and can mimic and replace solid fats due to the creation of new intermolecular hydrogen bonds which improve their gel strength and crystallinity (Sintang et al. 2017).

Adipic acid (AA) is a naturally occurring linear dicarboxylic acid found in beets and sugarcane. It is a biocompatible and non‐toxic compound widely used in the food industry as a safe food additive (E355), which serves various purposes such as flavoring, acidity regulation, and gelling. Previous research suggests that adipic acid plays an essential role in the nucleation and crystallization process (Adili et al. 2020). Beeswax is a complex mixture containing over 300 components, including hydrocarbons, free fatty acids, fatty acid esters, fatty alcohol, and diesters (Fratini et al. 2016). It is a permitted food additive with numerous applications in the food industry, such as stabilizing, creating texture in chewing gum, carrying flavor compounds and colors, providing gloss, releasing agents, and acting as an organogelator. Numerous studies have investigated the use of beeswax as an organogelator in edible oils. Beeswax oleogels form needle‐like crystalline structures that are safe for human consumption (Singh et al. 2017).

Black seed, also known as *Nigella sativa* L., is a popular herbaceous plant from the Ranunculaceae family. The seeds of black seed contain a substantial amount of oil (28%–36%), which is a valuable nutrient with therapeutic and protective potential for human health (Dinagaran et al. 2016). Black seed oil is widely used as a food supplement due to its numerous health benefits, including antioxidant, anti‐inflammatory, anti‐cancer, antimicrobial, and digestive system protection properties (Suri et al. 2019). In addition to its high fatty acid content, black seeds also contain phenolic compounds, tocopherol, sterols, and polar lipids (Mazaheri et al. 2019). Black seed oil is rich in saturated fatty acids, mainly palmitic and stearic acids, as well as unsaturated fatty acids like oleic and linoleic acids (Gad and El‐Ahmady 2018). It is considered a important source of essential fatty acids and can be used as a fat substitute (Suri et al. 2019).

The aim of this research was to investigate the synergistic effect of beeswax and adipic acid on black seed oil oleogel formation. At the next step, the most suitable oleogel treatment with animal fat in the formulation of beef hamburgers was evaluated. The physical, chemical and sensorial characteristics of oleogel filled and animal fat reduced hamburgers were investigated.

## 2 Materials and Methods

### 2.1 Materials

Edible beeswax was acquired from Neutron Company (Iran) and adipic acid was obtained from Tokyo Sanat Co (Japan). Black seed oil was sourced from a local market in Urmia, Iran. The raw materials required for the preparation of hamburgers were also obtained from the local market in Urmia, Iran. The chemicals used in this study were obtained from Sigma Aldrich and Merck, Germany.

### 2.2 Oleogel Preparation

To prepare the black seed oil oleogels, a combination of beeswax and adipic acid was used in varying ratios (as indicated in Table 1) at a level of 5% w/w of edible oil, following the method of Tabibiazar et al. (2020) with minor modifications. The oleogels were produced by heating black seed oil to a temperature of 150°C (higher than the melting point of adipic acid and beeswax) with continuous stirring and gradual addition of the gelator. The mixture was stirred using a magnetic stirrer for 3 min until the compounds were completely dissolved in the oil phase. After thorough mixing, the oleogels were transferred to analysis containers and cooled in an ice water bath. The samples were then kept at 4°C and subjected to relevant analyses after 24 h. Prior to testing, the samples were allowed to reach room temperature for 30 min (Tabibiazar et al. 2020).

**TABLE 1:** Proportions of beeswax, adipic acid and black seed oil used for preparation of oleogels and their codes.

Treatments	Adipic acid (%)	Beeswax (%)	Black seed oil (95%)
A5	5	0	95
B5	0	5	95
B1A4	4	1	95
B4A1	1	4	95
B2A3	3	2	95
B3A2	2	3	95
B2.5A2.5	2.5	2.5	95

### 2.3 Oleogel Characterization

#### 2.3.1 Oil Binding Capacity

Oil binding capacity (OBC) was measured by the following procedure. Initially, the weight of empty microtubes was recorded (a). Next, 2 mL of melted oleogel samples were dispensed into the microtubes, which were then stored at room temperature for 24 h. The microtubes were then re‐weighed (b), followed by centrifugation at 1000 rpm for 15 min at room temperature. Excess oils were removed by centrifuging the microtubes for 3 min on filter paper, and the microtubes were weighed again (c). Finally, the OBC was determined using the following formula (Fayaz, Goli, and Kadivar 2017; Fayaz, Goli, Kadivar, Valoppi, et al. 2017):

$$\mathrm{OBC}\%=100-\left(\;\frac{\left(b-a\right)-\left(c-a\right)}{\left(b-a\right)}\times 100\right)$$

#### 2.3.2 Color Measurement

Approximately 5 g of oleogel samples were placed on a plate at room temperature, and their color parameters (L*, a*, b*) were measured using a Colorimeter (CR—400, Japan). The L* parameter indicates sample brightness, the a* parameter indicates color similarity to red and green, and the b* parameter indicates color similarity to blue and yellow.

#### 2.3.3 Texture Analysis

To assess the hardness of the oleogels, a penetration test was conducted at room temperature using a cylindrical probe with a diameter of 12 mm and a weight of 5 kg. The test involved applying a force of 17 g to the cylindrical containers at a speed of 100 mm/min, resulting in a penetration depth of 10 mm. The resulting deformation was analyzed using texture analysis (TA.XT Plus, Stable Micro Systems Ltd., Surrey, England) (Fayaz, Goli, and Kadivar 2017; Fayaz, Goli, Kadivar, Valoppi, et al. 2017).

#### 2.3.4 Crystal Morphology

A portion of the melted oleogels was poured onto a slide and the lamella was positioned at a 45‐degree angle. Crystallization was allowed to occur at room temperature for 24 h, and the resulting crystals were observed using a polarized optical microscope (Leica DM 2000, Switzerland) with a 40× magnification lens (Tabibiazar et al. 2020).

#### 2.3.5 Rheological Properties

The modulus of elasticity (G´) and viscosity (G´´) of the oleogel samples were measured using a Dynamic Rheometer (Anton PaarPhysica MCR 301, Graz, Austria) with a parallel plate probe at room temperature (25°C). Approximately 4 g of oleogel was placed in the container, and the probe was adjusted to a 1 mL sample. The modulus of elasticity and viscosity were measured by performing frequency scans ranging from 1 to 10 rad and applying a constant strain of 0.1% within the linear viscoelastic region (Sagiri et al. 2015).

#### 2.3.6 Thermal Properties

The thermal properties of the oleogel samples were analyzed using Differential Scanning Calorimetry (TA4000, Switzerland). An amount of 8 mg of oleogel sample was placed in a weighing aluminum container and sealed. The temperature range was set from 30°C to 300°C at a rate of 5°C/min under a nitrogen atmosphere (Tabibiazar et al. 2020).

#### 2.3.7 Fatty Acid Composition

A total of 7.5 g of weighed oleogel sample was mixed with a 1:1 (v/v) methanol‐chloroform mixture of 30 mL. The mixture was sonicated for 10 min, followed by the addition of 10 mL of distilled water and constant mixing to form three phases. The mixture was filtered through filter paper to remove solid particles and allowed to form two phases. To obtain better phase separation, the mixture was centrifuged for 3 min at 3500 rpm. The methanol phase was then separated from the oil and chloroform phases, and the chloroform solvent was evaporated. In order to determine the fatty acid composition, a sample of oil measuring approximately 50 μL was mixed with 100 μL of 0.5 N sodium methoxide in a vial. The mixture was then combined with 1 mL of hexane and thoroughly dissolved for 5 s using a mixer. The resulting phase was transferred to a container containing sodium sulfate for dehydration. The fatty acid methyl ester in this layer was then evaluated using gas chromatography (Agilent 6890 N, America) with a HP‐88 column measuring 100 m in length, 0.25 mm in outer diameter, and 0.2 mm in inner diameter. Nitrogen was used as a carrier gas, and the injection site was heated to 280°C before analysis. The carrier gas used for gas chromatography was nitrogen, and the injection site was heated to 280°C prior to analysis. The temperature program involved starting the column at 140°C for 5 min, followed by an increase of 4°C/min until it reached 240°C, and then holding it at that temperature for 25 min. After the methyl ester separation of fatty acids on the column, a flame ionization detector was used at 300°C, and hydrogen gas was used for detection at a flow rate of 300 mL/min. The type of fatty acids presented in the samples was identified based on different standards, and the percentage of each fatty acid in each sample was reported (Goli et al. 2013).

#### 2.3.8 Oxidative Stability of Oleogel

The oleogel samples were stored at refrigerator temperature for 30 days, and the peroxide index was measured using the AOAC standard. In this test, 5 g of oil was mixed with 30 mL of a 2:3 acetic acid‐chlorophorm solution. After 1 min of storage in a dark place, 30 mL of distilled water and a few drops of 1% starch were added, and the titration was performed using a normal sodium thiosulfate until the dark color disappeared. The same procedure was followed for the control sample without the addition of any oil sample.

$$\mathrm{PV}=\frac{\left(V2-V1\right)\times N\times 1000}{m}$$

In the aforementioned equation, V1 represents the volume of sodium thiosulfate used for the control sample, while V2 represents the volume of sodium sulfate used for the test sample. N stands for the normalization of sodium thiosulfate, and m represents the weight of the sample in grams.

### 2.4 Production and Physicochemical Analysis of Hamburgers

#### 2.4.1 Hamburger Production

The beef meat (82% lean beef and 18% fat) was obtained from a local supermarket. The meat was ground by using a mincer (Severin Elektrogeräte GmbH, Sundern, Germany). Then, 52% ground beef (lean beef), 18% animal fat, 12% grated onion, 12% soy, 4% wheat flour, 1% salt and pepper and 1% gluten were mixed completely (Hemmatkhah et al. 2020). The mixed materials were shaped into hamburger samples and stored in the freezer. The samples had an overall weight of 100 g, a thickness of 0.5 cm and a diameter of 11 cm. The samples were then stored in the freezer and evaluated on days 1, 15 and 30. To assess the characteristics of the fried hamburgers, they were fried at 110°C for 5 min using a fryer. Based on the results, the most suitable oleogel treatment was the treatment containing 5% beeswax. This treatment was employed as a substitute for animal fat in the formulation of beef burgers. The control treatment comprised 18% total fat in the final product. Three experimental treatments were investigated, wherein 6%, 9%, and 12% of the total fat content was replaced with beeswax oleogel, respectively.

#### 2.4.2 Cooking Loss

The measurement of cooking loss was performed using the following protocol. Three hamburger slices were selected from both the control treatment and samples containing oleogel. These slices were weighed using a digital scale. The slices were then fried in sunflower oil for 5 min at a temperature of 110°C. After frying, the slices were weighed again, and the percentage of cooking loss was calculated using the following equation (Akwetey and Knipe 2012):

$$\text{Cooking Loss}\%=\frac{M1-M2}{M1}\times 100$$

where M1 represents the raw weight of the hamburger, while M2 represents the weight of the cooked hamburger.

#### 2.4.3 Moisture Retention

To measure moisture retention, the moisture content of both the control sample and samples containing oleogel were determined before and after cooking using the AOAC (1995) standard. The percentage of moisture retention in the hamburger samples was then calculated using the following formula.

$$\text{Moisture retention}\%=\frac{M1}{M2}\times 100$$

where M1 represents the moisture content of the cooked hamburger, while M2 represents the moisture content of the raw hamburger.

#### 2.4.4 Fat Absorption

The fat absorption rate was measured by first drying the raw and cooked hamburger samples to remove any moisture. The amount of fat in the hamburgers before and after frying was then determined using the Soxhlet method (BüCHI Extraction System B‐811). The amount of fat absorbed by the samples was calculated using the following equation (Soltanizadeh and Ghiasi‐Esfahani 2015).

$$\mathrm{Fat}\;\text{Absorption}\%=\left(M2-M1\right)\times 100$$

In the aforementioned equation, M1 represents the amount of raw hamburger fat, while M2 represents the amount of cooked hamburger fat.

#### 2.4.5 Color Measurement

The colorimetry involved measuring the color parameters (L*, a*, b*) of both the raw and cooked samples using a Hunter Lab device (CR—400, Japan). For this purpose, 15 g of hamburger samples were separated and shaped into circles, and then the machine was calibrated using a standard white tile for the machine, and finally, the color indices of the hamburger samples were measured.

#### 2.4.6 Diameter Reduction or Shrinkage

The measurement of diameter reduction or shrinkage involved selecting three hamburgers from both the control sample and the samples containing oleogel. The diameter of the hamburgers was measured before and after frying using a caliper, and the percentage of diameter reduction in the hamburger samples was then calculated using the following formula (Modi et al. 2004).

$$\text{Diameter reduction}\%=\frac{D1-D2}{D1}\times 100$$

In the aforementioned equation, D1 represents the diameter of the raw hamburger, while D2 represents the diameter of the cooked hamburger.

#### 2.4.7 Hamburger Oxidation Rate

The rate of hamburger oxidation was determined by measuring the thiobarbituric acid index. To achieve this, 20 g of hamburger sample was mixed with 50 mL of 20% weight/volume trichloroacetic acid (TCA) for 2 min using a mixer. The contents of the mixer were then washed with 50 mL of distilled water and filtered through filter paper. Next, 5 mL of the filtered solution was mixed with 5 mL of 0.01 M thiobarbituric acid reagent and left in a bain‐marie for an hour. The absorbance of the pink solution was then measured at a wavelength of 532 nm, multiplied by a factor of 5.4, and used to calculate the mg of malonaldehyde/kg of sample (Soltanizadeh and Ghiasi‐Esfahani 2015).

#### 2.4.8 Texture Profile Analysis

The texture of the hamburgers, particularly the level of cohesion before and after frying, was evaluated using a texture measuring device (TA.XT Plus, Stable Micro Systems Ltd., Surrey, England). A 2 cm diameter and 1 cm thick sample was prepared from each hamburger, and both raw and fried samples were compressed at room temperature up to 50% of their height using a two‐stage mechanism (TPA) with a 5 kg load cell. The compression was carried out at a constant speed of 2 mm/s, and the maximum force required for compression was recorded as an indication of the overall texture difficulty.

#### 2.4.9 Sensory Evaluation

Sensory analysis was performed by 20 semi‐trained panelists using five‐point hedonic scale. To perform the test, 3 cm thick slices were prepared from the samples and randomly placed in plates made of disposable containers. These plates were without color and smell and were numbered. The evaluated characteristics included taste, aroma, color, texture (softness and firmness), and overall acceptance.

### 2.5 Statistical Analysis

All experiments were conducted in a randomized design and data were analyzed using one‐way analysis of variance (ANOVA) in SPSS22 software. The tests were repeated three times, except for the device tests. Duncan's test was employed at a 95% probability level (*p* < 0.05) to determine the significant differences among means. Graphs were plotted using Excel (2016) and Origin2021 software.

## 3 Results and Discussion

### 3.1 Evaluation of Oleogel Properties

#### 3.1.1 Texture Evaluation of Oleogels

**FIGURE 1:**
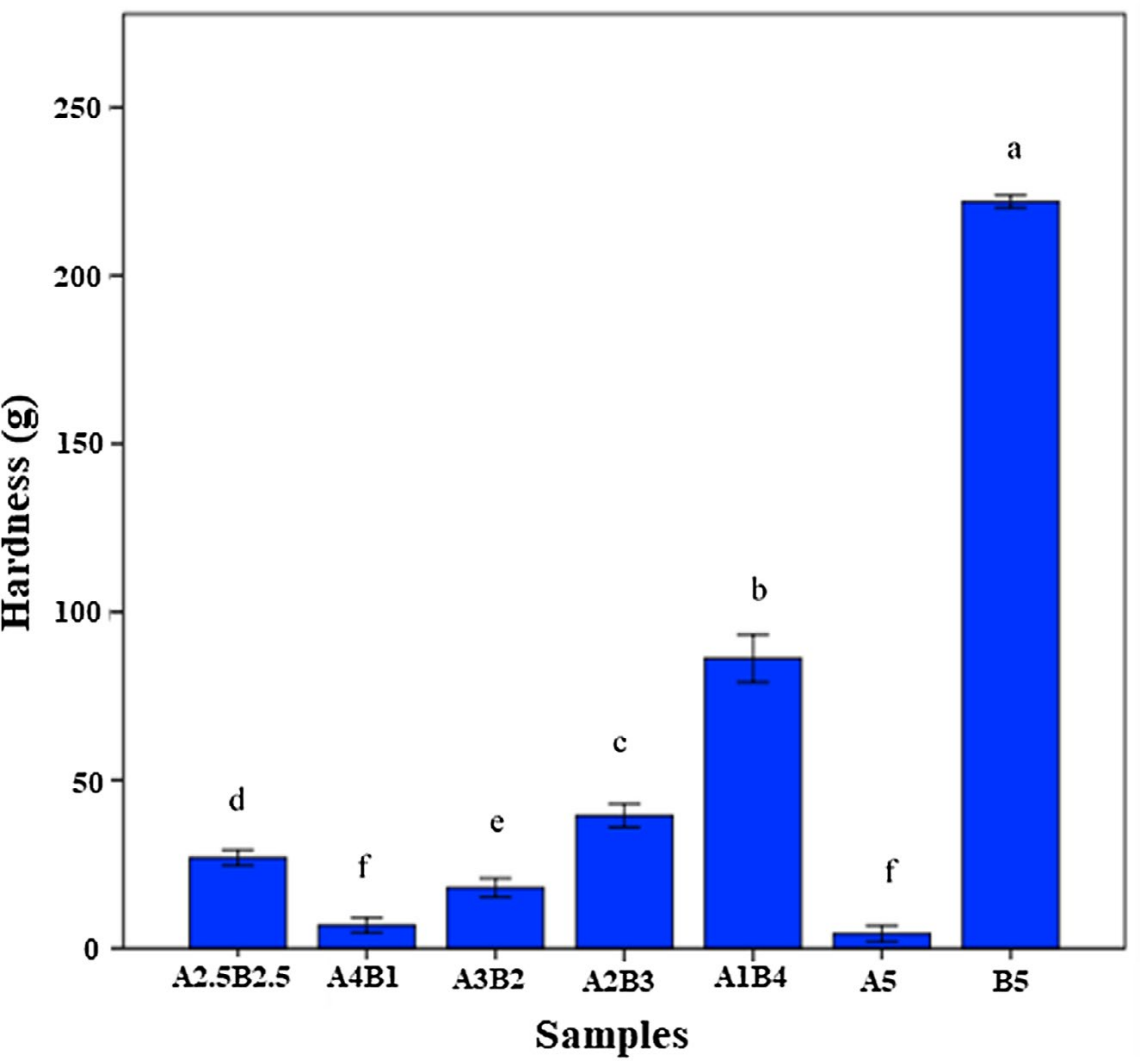
Hardness values of different black seed oil oleogels prepared by 5% w/w gelator. Lowercase letters denote a significant difference at the 5% level of Duncan's test (*p* < 0.05).

Texture is a crucial factor in evaluating oleogels, and hardness is the most important histological factor for measuring it. The hardness is referred to as the maximum force necessary to cause a permanent change in texture. Figure 1 shows the texture hardness of oleogels with different proportions of beeswax and adipic acid. Based on the results, pure beeswax oleogel (B5) displayed the highest hardness, while pure adipic acid oleogel (A5) showed the lowest hardness among the samples. Among the combined oleogels, A1B4 oleogel showed the highest level of hardness, although its level was still lower than that of pure beeswax oleogel.

The hardness of the combined oleogels can be significantly increased (*p* < 0.05) by increasing the concentration of beeswax and decreasing the concentration of adipic acid. This is due to the fact that beeswax crystals have greater cohesion than adipic acid in black seed oil. However, when replacing some beeswax with adipic acid in the preparation of black seed oil oleogel, there was no increase in texture hardness compared to using pure beeswax. Therefore, the combination of beeswax and adipic acid in the preparation of black seed oil oleogel did not have a synergistic effect on texture hardness. Winkler‐Moser et al. (2019) observed similar results when using a combination of beeswax and sunflower wax to prepare soybean oil oleogel. The combined oleogels had less hardness than the oleogels obtained from pure wax, indicating that the combination of these two waxes did not show a synergistic effect on the hardness of the oleogels (Winkler‐Moser et al. 2019). Callau et al. (2020) examined the hardness of sunflower oil oleogels created by combining benyl alcohol and behenic acid in different 10% w/w ratios. The results revealed that the 7:3 ratio of benyl alcohol to behenic acid produced the hardest oleogel, indicating a synergistic effect between the gelators. Conversely, the oleogel created from pure behenic acid had the lowest hardness value among all the samples. These findings are consistent with the current research, which found that adipic acid had the lowest level of hardness among all the samples.

#### 3.1.2 Crystal Morphology

**FIGURE 2:**
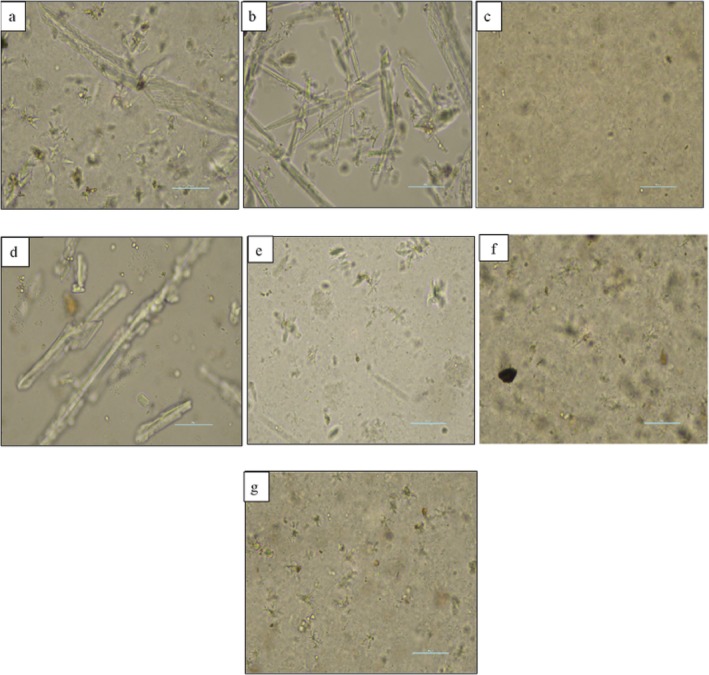
Crystal images of black seed oil oleogels prepared by 5% w/w gelator ((a) A2.5B2.5; (b) A5; (c) B5; (d) A4B1; (e) A3B2; (f) A1B4; (g) A2B3).

The morphology of crystals in black seed oil oleogels was observed using a polarized light microscope, and the resulting images are presented in Figure 2. The crystals in oleogel B5 (Figure 2c) were found to be needle‐shaped and coherent, consistent with the findings of Moghtadaei et al. (2018). Increasing the concentration of beeswax in the oleogels resulted in smaller needle‐shaped crystals and stronger, stiffer oleogels. In contrast, oleogel A5 (Figure 2b) exhibited large and irregular crystals, similar to the observations made by Tabibiazar et al. (2020), although their report indicated the formation of more and narrower crystals. The crystal structure of A5 was also evident to some extent in samples A4B1 (Figure 2d), A2.5B2.5 (Figure 2a), and A3B2 (Figure 2e). When the concentration of adipic acid in the combined oleogel formula was less than 3%, needle‐shaped beeswax crystals were dominant due to beeswax's superior ability to trap oil compared to adipic acid.

#### 3.1.3 Oil Binding Capacity (OBC)

The OBC is a critical parameter for oleogel application in food products, as it indicates the amount of oil trapped in the three‐dimensional network by the gelator (Pandolsook and Kupongsak 2017). Oleogels can hold up to 99% oil, requiring a high OBC to prevent oil leakage (Blake et al. 2014). Table 2 indicates that the OBC of the samples ranges from 25.48 to 100%, with sample B5 exhibiting the highest value and sample A5 showing the lowest value. The OBC of the samples increased with decreasing percentage of adipic acid in the oleogel composition, with these differences being statistically significant (*p* < 0.05). These findings are consistent with the results of Adili et al.'s (2020) study, in which the pure adipic acid oleogel had the lowest oil binding capacity among other samples. In a study conducted by Gao et al. (2021) to investigate the impact of cooling temperature (4°C and 25°C) and various concentrations of beeswax (2.5, 5, 7.5, 10, 12.5) on the OBC of oleogels made from rapeseed oil, it was found that the OBC of all samples was 100%, regardless of the variables. This finding is in agreement with the results of a previous study by Yılmaz and Öğütcü (2014), which evaluated the OBC of olive oil oleogels made with beeswax and sunflower wax gelators and found that the OBC of all samples was above 99%. These findings indicate that wax‐based oleogels, especially those made with beeswax, exhibit high stability and oil binding capacity, making them suitable for use in food products.

**TABLE 2:** Storage capacity of oleogel oils at 5% w/w gelator and a temperature of 25°C.

Samples	OBC%
A5	25.48 ± 0.39^c^
A4B1	30.3 ± 0.83^c^
A3B2	48.12 ± 0.65^b^
A2.5B2.5	99.96 ± 0.03^a^
A2B3	99.96 ± 0.03^a^
A1B4	99.98 ± 0.06^a^
B5	100 ± 0.00^a^

*Note:* Reported data are as mean ± standard deviation (*n* = 3). Non‐common lowercase letters denote a significant difference at the 5% level of Duncan's test (*p* < 0.05).

#### 3.1.4 Color Evaluation

Color properties play a crucial role in determining the suitability of oleogels for food industry applications. Table 3 presents the color evaluation results for black seed oil oleogels. Among the samples, oleogel A5 exhibited the highest brightness L*, while A2B3 showed the lowest. The decrease in beeswax concentration and increase in adipic acid concentration in combined oleogels leads to a significant increase in L* (*p* < 0.05), which can be attributed to greater light scattering by the crystal network. Moreover, higher adipic acid concentration increased the redness index a* in the samples, as confirmed by Tabibiazar et al.'s (2020) research. A2B3 had the lowest, while A5 had the highest yellowness b* among the samples. Additionally, an increasing trend was observed in b* index with concentrations of adipic acid exceeding 2% in combined oleogels. In contrast to findings that carnauba wax oleogel exhibited the highest degree of yellowness and adipic acid oleogel the lowest, this study indicates that beeswax does not have a positive impact on increasing the yellowness index. Instead, an increase in adipic acid concentration significantly elevated this index (*p* < 0.05). Moghtadaei et al.'s (2018) research on beeswax‐derived oleogels showed that as the beeswax concentration rises, the yellowness and brightness intensities increase while the green intensity decreases. This finding contradicts this study, possibly due to variations in the oil type. Overall, all oleogels possess a dark color that tends towards black, which may be attributed to the black seed oil's color. As a result, the oleogels' color is somewhat dependent on the oil type and gelator concentration, as indicated by previous research.

**TABLE 3:** Color index of oleogels at 5% w/w gelator.

Samples	Color parameters		
	b*	a*	L*
A5	9.16 ± 0.27^a^	1.62 ± 0.17^a^	37.02 ± 0.1^a^
A4B1	5.66 ± 0.32^b^	−1.25 ± 0.15^b^	28.72 ± 0.52^b^
A3B2	5.63 ± 0.23^b^	−1.23 ± 0.27^b^	27.61 ± 0.35^c^
A2.5B2.5	3.23 ± 0.05^c^	−1.21 ± 0.02^b^	26.43 ± 0.48^d^
A2B3	2.68 ± 0.08^d^	−1.6 ± 0.03^c^	25.53 ± 0.21^e^
A1B4	3.38 ± 0.38^c^	−1.51 ± 0.19^bc^	25.88 ± 0.8^de^
B5	3.04 ± 0.01^cd^	−1.39 ± 0.13^bc^	26.61 ± 0.45^d^

*Note:* Reported data are based on mean ± standard deviation (*n* = 3). Non‐common lowercase letters denote a significant difference at the 5% level of Duncan's test (*p* < 0.05).

#### 3.1.5 Thermal Characteristics

**FIGURE 3:**
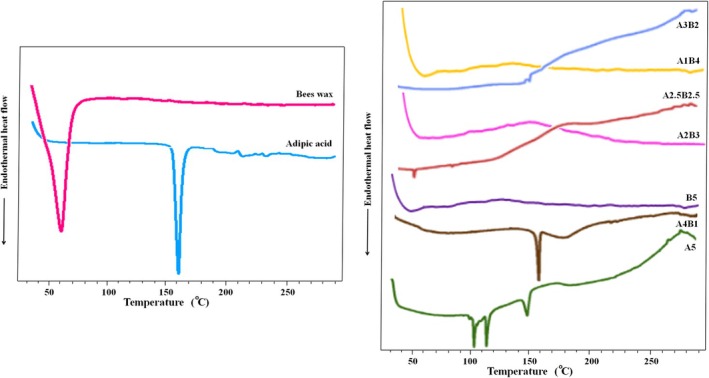
The DSC thermograms of adipic acid powder, beeswax, and black seed oil oleogels prepared by 5% w/w gelator at different proportions of beeswax and adipic acid.

The thermal characteristics of adipic acid powder, beeswax, and various oleogels at a 5% w/w level were evaluated and presented in Figure 3. The powder of adipic acid exhibited a melting temperature peak at 150°C, while beeswax showed a peak at 61°C. Thermograms of the oleogels revealed that the beeswax‐derived oleogel (B5) had a minor peak at 50°C that corresponded to the melting point. The lower concentration and dissolution of wax in liquid oil led to the reduced melting temperature of wax in oleogel relative to pure wax (Blake et al. 2014). The oleogel obtained from adipic acid demonstrated the same thermal behavior as adipic acid powder, and its corresponding peak disappeared in oleogels as the concentration of adipic acid decreased. In the oleogel containing equal proportions of adipic acid and beeswax (A2.5B2.5), a tiny and sharp peak was observed, likely related to the melting temperature of beeswax in oleogel. In general, none of the thermograms obtained from the oleogels exhibited both peaks related to beeswax and adipic acid. Instead, all combined oleogels displayed a single peak corresponding to one of the gelators, and no peak related to either gelator was observed in the B3A2 oleogel. This absence of peaks may be due to the mutual reaction of gelators in this oleogel. Additionally, oleogels with concentrations greater than 3% adipic acid had higher melting temperatures than other samples.

#### 3.1.6 Rheological Characteristics

**FIGURE 4:**
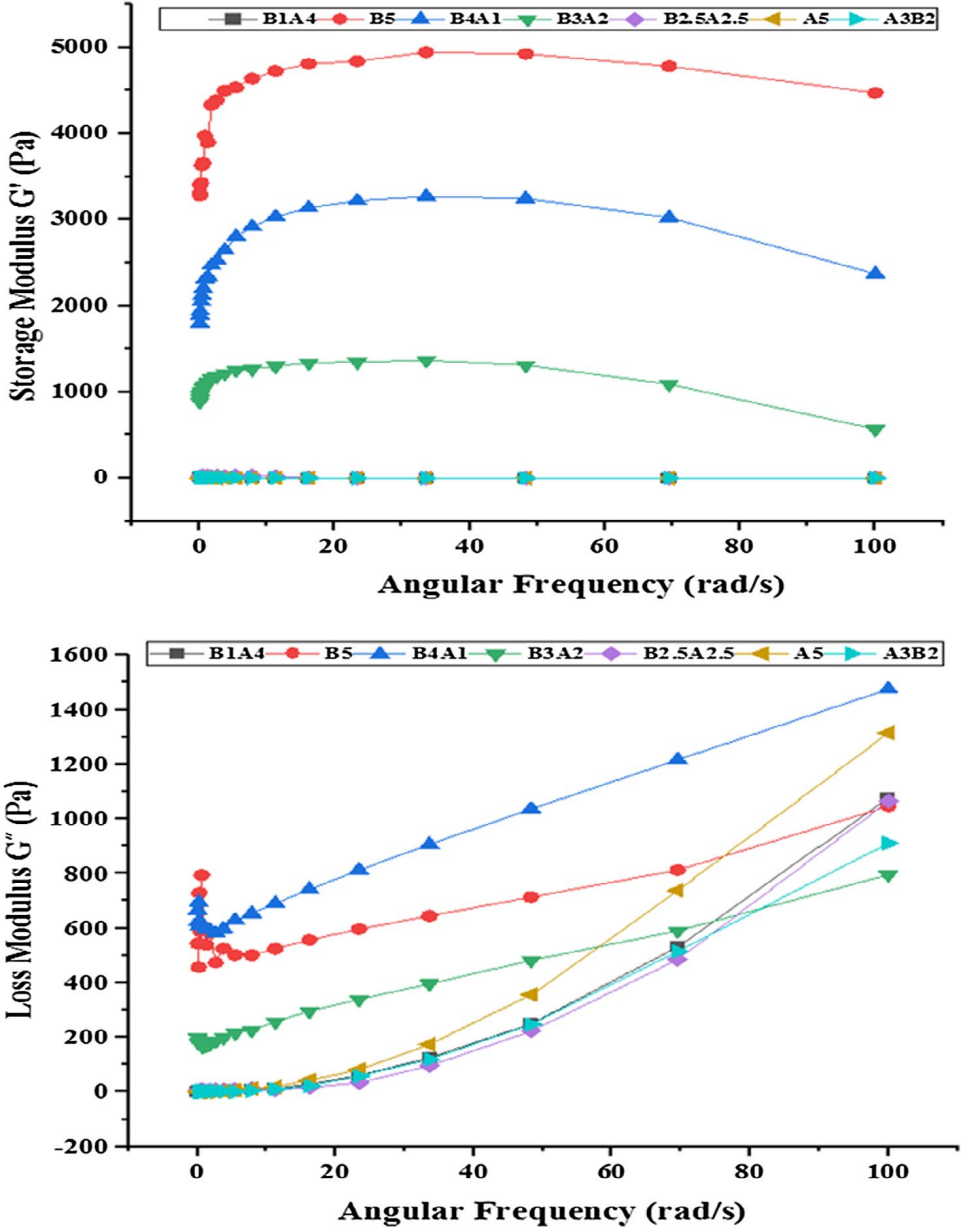
Rheological characteristics (storage modulus and loss modulus) of black seed oil oleogels prepared by 5% w/w gelator and recorded at a frequency range of 0.1–100 rad/s at 25°C.

The evaluation of rheological characteristics was conducted using a frequency scan test in the range of 0.1–100 rad/s, and the results are presented in Figure 4. The elastic modulus (G′) and the viscous modulus (Gʺ) represent the solid and quasi‐liquid components, respectively. A substance is considered a gel if its elastic modulus is higher than the viscous modulus. The results indicate that samples B5, B4A1, and B3A2 had significantly higher elastic moduli compared to other samples. Oleogels containing adipic acid initially exhibited a higher elastic modulus than the viscous modulus. However, for frequencies above 20 rad/s, the Gʺ became higher than the G′, indicating the weak structure of these oleogels. Sample A5 demonstrated the highest degree of frequency dependence, with an increasing viscous modulus with increasing frequency, confirming the weak structure of this oleogel. Blake et al. (2018) reported that a higher elastic modulus is often linked to the existence of smaller crystals that establish a uniform and homogeneous structure (Blake et al. 2018). This study also confirms this assertion. The results from this test are generally consistent with the findings from the histometric test, indicating that the combination of beeswax gelators and adipic acid did not lead to a synergistic effect on the rheological characteristics of black seed oil oleogels. In a study by Fayaz, Goli, and Kadivar (2017), Fayaz, Goli, Kadivar, et al. (2017) that utilized a combination of beeswax and propolis in pomegranate seed oil oleogel, it was found that this combination of gelators did not result in a positive effect on increasing the elastic modulus of oleogels. Instead, the oleogel obtained from beeswax demonstrated the highest elastic modulus compared to other samples, which is similar to the findings of this study. In contrast, Adili et al. (2020) conducted a study on the combination of ethyl cellulose and adipic acid gelators with different ratios at a concentration of 6% w/w and found that these two gelators in EC2AA4 oleogel had a synergistic effect on rheological properties, contradicting the results of this study.

#### 3.1.7 Fatty Acid Profile

Table 4 displays the fatty acid profile of black seed oil and the oleogel containing 5% beeswax (B5), which exhibited the best physical and chemical properties. The unsaturated fatty acids make up 77.88% of black seed oil, with linoleic acid and oleic acid being the most abundant. Palmitic acid is the most prevalent saturated fatty acid. Comparison of the fatty acid composition of black seed oil and the beeswax oleogel indicated that the oleogel manufacturing process had little impact on the fatty acid composition. The fatty acid composition of sesame oil and oleogels containing 2.5%, 7.5%, and 10% beeswax was similar, and the production process did not affect the composition of fatty acids. Black seed oil oleogel, with lower percentages of myristic acid (8.48%), palmitic acid (28.37%), and stearic acid (8%) compared to fat (Moghtadaei et al. 2018), can be a healthier substitute in hamburger formulations for ensuring consumer health.

**TABLE 4:** Fatty acid profile of black seed oil and the most suitable oleogel sample (B5).

Samples	The percentage of fatty acids							
	Myristic	Palmitic	Stearic	Behenic	Palmitoleic	Oleic	Linoleic	Alpha‐linolenic
Black seed oil	0.27	12.68	4.71	4.07	0.32	27.77	49.18	0.61
Oleogel B5	0.46	13.89	4.47	4.01	0.36	27.51	47.03	1.58

#### 3.1.8 Evaluation of Peroxide Value

**FIGURE 5:**
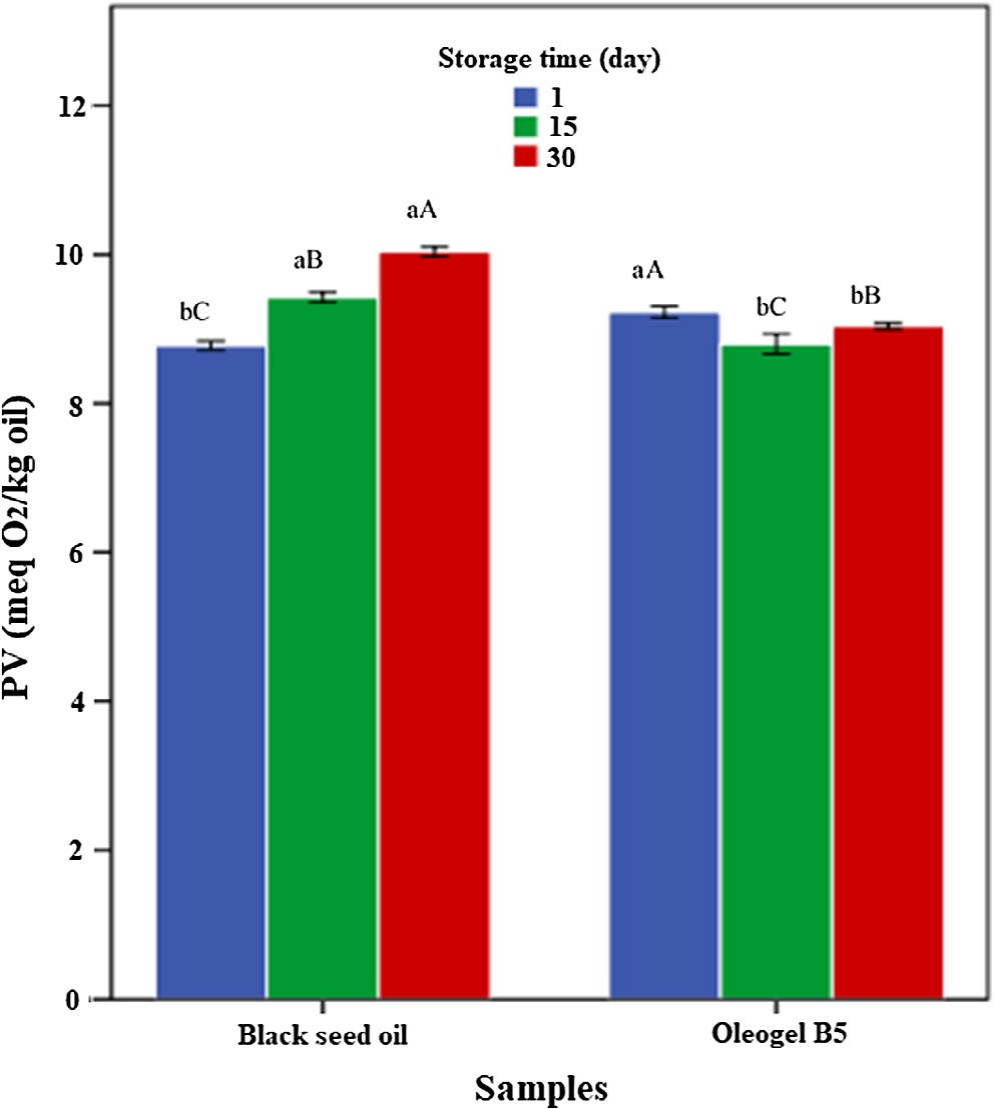
The peroxide value (PV) of the most suitable oleogel sample (B5) and black seed oil after 30 days of storage. Lowercase letters denote a significant difference at the 5% level of Duncan's test (*p* < 0.05) between the samples. Uppercase letters denote a significant difference at the 5% level of Duncan's test (*p* < 0.05) between the times.

Oxidation is a crucial reaction in oils and fats that significantly impacts product quality. Hydroperoxides, the primary oxidation products, are measured through the peroxide index. The results of peroxide value of the best oleogel sample (B5) and black seed oil after 30 days of storage are illustrated in Figure 5. Initially, the optimal oleogel sample showed a higher peroxide value than black seed oil. The peroxide value of black seed oil increased significantly over time (*p* < 0.05). However, in the optimal oleogel sample, it decreased after 15 days, increased again on the 30th day, and the increase level was lower than the control sample. The high initial peroxide content of the oleogel sample compared to black seed oil may be due to the effects of temperature and stirring during the oleogel production process. Lim et al. (2017) reported that continuous stirring of oil and gelator at high temperatures during oleogel preparation can negatively affect oil oxidation. They also found that the hardness of the oleogel sample is inversely related to peroxide content during storage. Furthermore, limiting oil migration and increasing oil storage capacity in oleogels can delay oil oxidation during storage (Lim et al. 2017). These findings are consistent with the results of this study, as oleogel B5 has a solid texture and shows less peroxide value increment in comparison to black seed oil, which is in liquid form. The lower peroxide content of the oleogel can also be partly attributed to the antioxidant properties of beeswax.

### 3.2 Evaluation of Hamburger Characteristics

#### 3.2.1 Moisture Retention

Maintaining proper moisture in hamburger is crucial for ensuring its appearance and taste. The moisture retention of hamburger samples prepared using beeswax oleogel (B5) with varying replacement percentages during storage time was evaluated and the results are presented in Table 5. The findings indicated that the moisture retention rate decreased significantly with time in both the control sample and samples containing oleogel (*p* < 0.05). Furthermore, the percentage of moisture retention was impacted by different replacement percentages, with an increase observed as replacement percentage increased (*p* < 0.05). The results also demonstrate that among all the samples, the sample containing 12% beeswax oleogel exhibited the highest moisture retention during storage, while the control sample displayed the lowest retention. A study conducted by Moghtadaei et al. (2018) on the replacement of sesame oil oleogel in beef hamburger showed that the hamburger containing 50% beeswax oleogel had higher moisture retention compared to the control sample and the sample containing 25% oleogel, which is consistent with this study's findings. Additionally, a study conducted by Özer and Çelegen (2021) on beef hamburger containing olive oil oleogel reported that the hamburger containing olive oil oleogel had higher moisture retention than the control sample (Özer and Çelegen 2021). Beeswax acted as a barrier to moisture loss during cooking and increased the water holding capacity of the hamburger samples, ultimately leading to a decrease in oil absorption during cooking and promoting the health of consumers.

**TABLE 5:** Moisture retention of hamburger samples prepared with beeswax oleogel (B5) at different replacement levels.

Samples	Storage time (day)		
	1	15	30
Control	96.44 ± 0.37^cA^	94.52 ± 0.64^cB^	89.17 ± 0.65^cC^
9% oleogel	98.42 ± 0.41^abA^	96.78 ± 0.32^abB^	95.44 ± 0.33^aC^
6% oleogel	97.49 ± 0.45^bA^	95.82 ± 0.73^bB^	92.18 ± 0.61^bC^
12% oleogel	99.18 ± 0.74^aA^	97.70 ± 0.80^aB^	95.62 ± 0.33^aC^

*Note:* Reported data are based on mean ± standard deviation (*n* = 3). Non‐common lowercase letters denote a significant difference at the 5% level of Duncan's test (*p* < 0.05) between the samples. Non‐common uppercase letters denote a significant difference at the 5% level of Duncan's test (*p* < 0.05) between the times.

#### 3.2.2 Color Evaluation

Evaluation of color and appearance is crucial in determining consumer acceptance of meat products. The color characteristics of hamburger samples during freezer storage are presented in Table 6. There was no significant difference in brightness L* of the control sample in both raw and cooked states over time (*p* < 0.05). However, in the raw samples containing 9 and 12% oleogel, the brightness significantly decreased over time. Meanwhile, the brightness of the raw sample containing 6% oleogel, similar to the control sample, did not show a significant difference over time. In the cooked sample containing 12% oleogel, there was no significant difference in this index over time, whereas there was a significant difference in the cooked samples containing 6% and 9% oleogel, but no trend was observed (*p* < 0.05). Furthermore, the replacement percentage significantly affected the brightness index, as the brightness decreased with an increase in the percentage of oleogel substitution compared to the control sample. The control sample exhibited the highest level of brightness, whereas the sample containing 12% oleogel had the lowest level. This difference could be attributed to the color of black seed oil. Regarding the degree of redness (a*), the raw hamburger without oleogel had the highest degree of redness at the beginning of storage, whereas the hamburger containing 12% oleogel had the lowest degree. As the replacement percentage increased, the redness of the samples decreased. This trend persisted on the 30th day of storage for raw samples. Overall, both storage time and replacement percentage had a significant effect on the redness index of raw samples (*p* < 0.05). In the cooked samples, the control sample exhibited the highest redness, whereas the sample containing 12% oleogel had the lowest. Over time, the degree of yellowness (b*) in the control sample and the samples containing 6 and 9% oleogel remained stable, while in the sample containing 12% oleogel, there was a significant decrease in yellowness intensity. At the beginning of storage, the intensity of yellowness was similar in the control sample and the samples containing 6 and 9% oleogel, while the sample containing 12% oleogel showed the least intensity of yellowness. During the storage period, the intensity of yellowness in all raw samples decreased significantly (*p* < 0.05). In the cooked samples, the control sample had the highest degree of yellowness, while the sample containing 12% oleogel had the lowest intensity of yellowness. Over time, there was a significant decrease in the intensity of yellowness in both the control sample and the sample containing 9% oleogel. However, the samples containing 6% and 12% oleogel did not exhibit significant changes on the 15th and 30th day. In contrast to this study, Tabibiazar et al. (2020) reported a significant increase in the yellowness of hamburgers containing carnauba wax oleogel with an increase in replacement percentage. Moghtadaei et al. (2018) reported that the decrease in brightness of beeswax oleogel could be attributed to the presence of heterogeneous environments with large and small fat globules that cause light scattering within the product, which is consistent with the results of this study. Additionally, Moghtadaei et al. (2018) reported that the intensity of yellowness and redness of the samples was not affected by fat replacement. Gómez‐Estaca, Herrero, et al. (2019), Gómez‐Estaca, Pintado, et al. (2019) conducted a study on the replacement of animal fat with oleogel in pork hamburgers and found that beeswax oleogel increased the yellowness of the samples compared to the control sample. However, in general, the replacement of black seed oil oleogel in hamburgers did not have a favorable effect on the color parameters, and in all cases, the control sample showed better changes than the substitute samples, which could be attributed to the use of green black seed oil.

**TABLE 6:** Color properties of raw and cooked hamburger samples prepared with different levels of substitution of honey bee wax oleogel (B5).

Samples	Type of hamburger and storage time (days)					
	Raw			Cooked		
	1	15	30	1	15	30
L*						
Control	52.86 ± 0.93^aA^	53.94 ± 0.03^aA^	53.50 ± 0.55^aA^	51.36 ± 1.04^aA^	50.66 ± 0.01^bA^	51.88 ± 0.13^aA^
9% oleogel	47.94 ± 0.63^cA^	46.36 ± 0.04^cB^	43.56 ± 0.01^dC^	45.94 ± 0.85^bA^	43.26 ± 0.01^dB^	42.11 ± 0.89^dB^
6% oleogel	50.95 ± 0.41^bA^	51.55 ± 0.22^bA^	51.20 ± 1.09^bA^	50.00 ± 1.35^aB^	52.11 ± 0.03^aA^	49.88 ± 0.04^bB^
12% oleogel	47.89 ± 0.13^cA^	46.41 ± 0.20^cB^	45.10 ± 0.14^cC^	44.81 ± 0.01^bA^	44.91 ± 0.03^cA^	44.67 ± 0.20^cA^
a*						
Control	5.75 ± 0.04^aA^	2.06 ± 0.04^aC^	4.14 ± 0.04^aB^	3.60 ± 0.01^aA^	3.37 ± 0.02^aB^	1.74 ± 0.02^aC^
9% oleogel	1.57 ± 0.06^cA^	0.82 ± 0.02^cB^	1.53 ± 0.03^bA^	0.84 ± 0.01^bA^	0.68 ± 0.02^bB^	0.48 ± 0.03^bC^
6% oleogel	1.86 ± 0.04^bA^	0.85 ± 0.01^bcC^	1.57 ± 0.02^bB^	−0.15 ± 0.01^cC^	0.18 ± 0.01^cB^	0.49 ± 0.01^bA^
12% oleogel	0.67 ± 0.01^dC^	0.89 ± 0.01^bB^	0.95 ± 0.01^cA^	−0.96 ± 0.01^dC^	−0.45 ± 0.02^dA^	−0.54 ± 0.03^cB^
b*						
Control	20.55 ± 0.45^aA^	19.28 ± 0.01^aB^	18.46 ± 0.02^aC^	22.76 ± 0.37^aA^	19.88 ± 0.02^aB^	15.76 ± 0.17^aC^
9% oleogel	20.61 ± 0.03^aA^	17.95 ± 0.03^dB^	16.67 ± 0.01^dC^	15.92 ± 0.10^bA^	13.26 ± 0.01^cB^	13.07 ± 0.02^cC^
6% oleogel	20.11 ± 0.35^aA^	18.72 ± 0.14^cB^	18.18 ± 0.01^bC^	16.28 ± 0.65^bA^	14.91 ± 0.01^bB^	15.53 ± 0.14^bAB^
12% oleogel	19.56 ± 0.40^bA^	18.96 ± 0.02^bB^	17.71 ± 0.01^cC^	13.65 ± 0.25^cA^	12.65 ± 0.01^dB^	12.91 ± 0.01^cB^

*Note:* Reported data are based on mean ± standard deviation (*n* = 3). Non‐common lowercase letters denote a significant difference at the 5% level of Duncan's test (*p* < 0.05) between the samples. Non‐common uppercase letters denote a significant difference at the 5% level of Duncan's test (*p* < 0.05) between the times.

#### 3.2.3 Cooking Loss

The cooking loss refers to the reduction in weight of a sample after frying compared to its raw weight. The results of the cooking loss of hamburger samples are presented in Table 7, indicating that both storage time and replacement percentage significantly impacted the cooking loss of the samples (*p* < 0.05). As shown in Table 7, the control sample exhibited the highest cooking loss, while the sample containing 12% oleogel had the lowest cooking loss. The increase in replacement percentage resulted in a decrease in cooking loss, which could be attributed to the improved moisture retention and water retention capacity of the samples due to beeswax oleogel. Furthermore, cooking loss increased in all samples with increasing storage time, with the control sample experiencing the highest increase and the sample containing 12% oleogel showing the least increase compared to the other samples. Based on the results, the replacement of black seed oil oleogel in hamburgers had a positive impact on reducing cooking loss. In a study by Oh et al. (2019) on the use of hydroxypropyl methyl cellulose oleogel as a fat substitute in meat pâtés, the use of this oleogel resulted in reduced cooking loss. Moghtadaei et al. (2018) also reported a reduction in cooking loss of hamburger samples through the use of beeswax oleogel. In another study conducted by the same researchers in 2021, they investigated the use of ethyl cellulose oleogel in beef hamburgers. The use of ethyl cellulose oleogel in hamburgers resulted in lower cooking loss compared to the control sample, as reported by Moghtadaei et al. (2021). Similar results were reported in a study by Gómez‐Estaca, Herrero, et al. (2019), Gómez‐Estaca, Pintado, et al. (2019) on the use of beeswax oleogel and ethyl cellulose oleogel in pâtés. Barbut et al. (2016) attributed the decrease in cooking loss in samples containing oleogel to the size of fat globules. They suggested that increasing the number of fat globules in the meat dough strengthens it, preventing moisture loss in meat products.

**TABLE 7:** Cooking loss (%) of hamburger samples prepared with different substitution levels of beeswax oleogels (B5).

Samples	Storage time (day)		
	1	15	30
Control	13.53 ± 0.03^aC^	15.72 ± 0.02^aB^	18.41 ± 0.04^aA^
9% oleogel	10.16 ± 0.05^cC^	11.89 ± 0.02^cB^	15.42 ± 0.04^cA^
6% oleogel	13.24 ± 0.04^bC^	14.82 ± 0.02^bB^	17.18 ± 0.02^bA^
12% oleogel	9.43 ± 0.03^cC^	11.06 ± 0.01^dB^	14.89 ± 0.04^dA^

*Note:* Reported data are based on mean ± standard deviation (*n* = 3). Non‐common lowercase letters denote a significant difference at the 5% level of Duncan's test (*p* < 0.05) between the samples. Non‐common uppercase letters denote a significant difference at the 5% level of Duncan's test (*p* < 0.05) between the times.

#### 3.2.4 Reduction in Hamburger Diameter

The reduction in hamburger diameter or shrinkage, which refers to a reduction in diameter, is an important quality parameter of hamburgers that occurs during cooking due to protein denaturation and the release of water and fat from the meat. The findings concerning the shrinkage of hamburger samples are presented in Table 8, which indicates that the control sample experienced the greatest degree of wrinkling, while the sample containing 12% oleogel had the least amount of shrinkage. The results demonstrated that the increase in replacement percentage leads to a significant decrease in sample wrinkling (*p* < 0.05). Additionally, the amount of wrinkling increased significantly with the passage of storage time, which could be due to the loss of moisture and protein denaturation over time. The findings of the current study are in agreement with those of Özer and Çelegen (2021), which showed that incorporating olive oil oleogel into composite samples reduced their shrinkage compared to the control sample. However, Moghtadaei et al. (2018) found that replacing animal fat with beeswax oleogel in hamburgers increased the wrinkling of oleogel‐containing burgers compared to the control burgers, which is in contrast to this research. This discrepancy could be due to differences in the type of oil used and the proportion of oleogel substitution. Moghtadaei et al. (2021) conducted a study on the application of ethyl cellulose oleogel in beef hamburgers and found that there was no significant difference in the degree of wrinkling between the hamburgers containing ethyl cellulose oleogel and the control sample. The findings regarding the reduction in diameter in this study are consistent with the results of the cooking loss measurements from the same study, as lower cooking loss typically results in less diameter reduction or wrinkling.

**TABLE 8:** Reduction in diameter (%) of hamburger samples prepared from beeswax (B5) with different substitution percentages.

Samples	Storage time (day)		
	1	15	30
Control	10.63 ± 0.46^aC^	13.93 ± 0.52^aB^	15.75 ± 0.52^aA^
9% oleogel	6.02 ± 0.05^bcB^	10.13 ± 0.90^bA^	10.94 ± 0.96^bA^
6% oleogel	6.96 ± 0.04^bC^	8.45 ± 0.47^cB^	10.12 ± 0.10^bcA^
12% oleogel	5.14 ± 0.03^cC^	6.79 ± 0.47^dB^	9.36 ± 0.46^cA^

*Note:* Reported data are based on mean ± standard deviation (*n* = 3). Non‐common lowercase letters denote a significant difference at the 5% level of Duncan's test (*p* < 0.05) between the samples. Non‐common uppercase letters denote a significant difference at the 5% level of Duncan's test (*p* < 0.05) between the times.

#### 3.2.5 Hamburger Oil Absorption

Table 9 presents the findings regarding the oil absorption of hamburgers following frying. The control sample demonstrated the highest oil absorption, while the sample containing 12% oleogel had the lowest oil absorption post‐frying. The results indicated that there was a significant reduction in the oil absorption of hamburger samples as the percentage of oleogel replacement increases (*p* < 0.05). Additionally, the oil absorption of all samples increased significantly as the storage time elapsed. During the frying process, moisture loss from hamburger samples leads to the creation of empty spaces that are filled by oil. It was observed that the samples containing oleogel experienced less moisture loss during frying compared to the control sample, resulting in a lower oil absorption rate. These findings align with those reported by Moghtadaei et al. (2018) and were consistent in another study conducted by the same researchers in 2021 (Moghtadaei et al. 2021). The reduction in oil absorption, combined with higher amounts of unsaturated fatty acids, has a significant impact on the nutritional quality of the hamburger and improves its beneficial effects on the health of consumers.

**TABLE 9:** Oil absorption (%) of hamburger samples prepared from beeswax (B5) with different substitution percentages.

Samples	Storage time (day)		
	1	15	30
Control	3.50 ± 0.04^aC^	3.76 ± 0.04^aB^	4.68 ± 0.05^aA^
9% oleogel	0.71 ± 0.05^cC^	2.58 ± 0.07^bB^	3.03 ± 0.02^cA^
6% oleogel	2.18 ± 0.02^bC^	2.81 ± 0.04^cB^	3.30 ± 0.06^bA^
12% oleogel	0.45 ± 0.06^dC^	1.92 ± 0.06^dB^	2.31 ± 0.06^dA^

*Note:* Reported data are based on mean ± standard deviation (*n* = 3). Non‐common lowercase letters denote a significant difference at the 5% level of Duncan's test (*p* < 0.05) between the samples. Non‐common uppercase letters denote a significant difference at the 5% level of Duncan's test (*p* < 0.05) between the times.

#### 3.2.6 Thiobarbituric Acid Index (TBARS)

**FIGURE 6:**
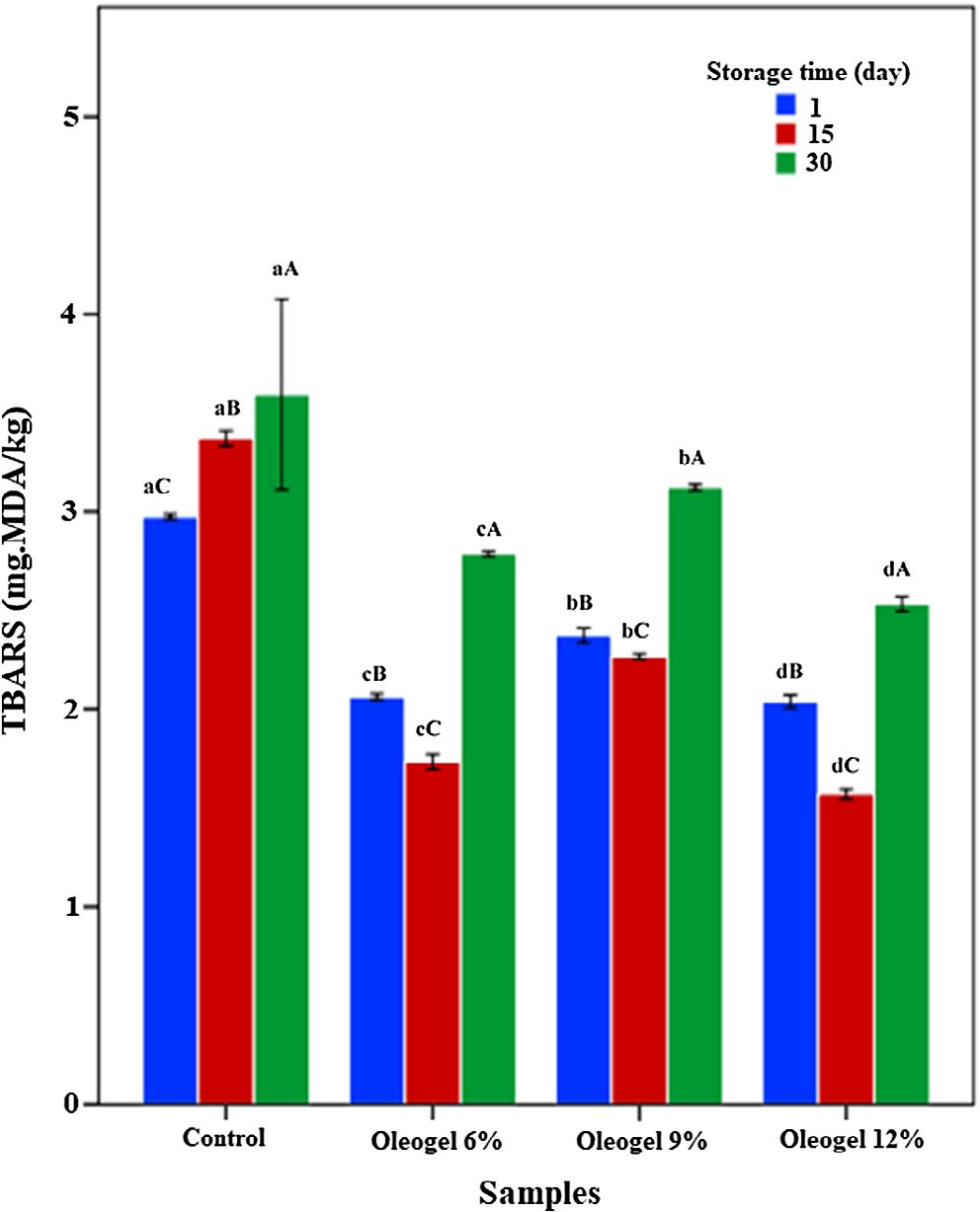
TBARS values of hamburger samples prepared from beeswax oleogel (B5) with various replacement percentages during freezer storage (−18°C). Lowercase letters denote a significant difference at the 5% level of Duncan's test (*p* < 0.05) between the samples. Uppercase letters denote a significant difference at the 5% level of Duncan's test (*p* < 0.05) between the times.

The TBARS index is commonly used to measure the degree of lipid oxidation in foods by analyzing secondary oxidation products. An increase in TBARS levels during storage can result in alterations to the sensory characteristics and color of meat‐based products. Figure 6 displays the TBARS index of hamburger samples stored in a freezer at −18°C. Based on the findings, the TBARS index increased significantly in the control sample over time. However, in the other samples, the index decreased after 15 days, which may be attributed to the antioxidant properties of black seed oil. Nonetheless, an increasing trend was observed again after 30 days. These outcomes are consistent with those reported in the study conducted by Özer and Çelegen (2021). The substitution of black seed oil in hamburgers had a positive impact on the oxidation stability of the samples in comparison to the control sample. Besides the type of oil, the quantity of phenolic compounds presented in beeswax and its antioxidant role may also contribute to the oxidation stability observed in samples containing oleogel relative to the control sample.

#### 3.2.7 Texture Profile Analysis (TPA)

The TPA results for this research focused on hardness, which is a critical factor that influences consumers' preference for meat products. Difficulty was the index used to evaluate this parameter, which indicates the maximum force needed for compression. The findings related to this parameter are presented in Table 10. The control sample showed a significant increase in difficulty over time (*p* < 0.05), while samples containing oleogel did not exhibit a consistent upward or downward trend. On the first day of storage, the sample with 12% oleogel had the softest texture, and the control sample had the hardest texture. The data suggests that samples containing oleogel have a less firm texture than the control sample. As a result, the raw samples' hardness significantly decreased with an increase in oleogel replacement percentage. For the cooked samples, on the first day of storage, the control sample had the highest hardness, while the sample with 12% oleogel had the lowest hardness. With an increase in replacement percentage, the hardness of the baked samples decreased significantly after 30 days. In a study by Moghtadaei et al. (2018), the use of beeswax oleogel in hamburgers resulted in an increase in the raw and cooked samples' hardness compared to the control sample. The authors attributed the cooked sample's hardness increase to oleogel weakening due to heat and oil leakage from the hamburger (Özer and Çelegen 2021). However, these findings contradict the results of this study, which could be due to differences in oil type, replacement percentages, and sample cooking conditions. Overall, the results indicate that the samples containing oleogel were more appealing to the panelists in terms of texture.

**TABLE 10:** Hardness (kg) of raw and cooked hamburger samples prepared from beeswax (B5) with different substitution percentages.

Samples	Type of hamburger and storage time (day)					
	Raw			Cooked		
	1	15	30	1	15	30
Control	0.08 ± 0.005^aB^	0.08 ± 0.005^cB^	0.13 ± 0.021^aA^	0.36 ± 0.004^bC^	0.43 ± 0.007^bB^	0.57 ± 0.069^aA^
9% oleogel	0.07 ± 0.001^bA^	0.07 ± 0.011^cA^	0.06 ± 0.024^bB^	0.33 ± 0.005^cA^	0.26 ± 0.015^dB^	0.26 ± 0.020^cB^
6% oleogel	0.08 ± 0.005^aC^	0.10 ± 0.005^aA^	0.09 ± 0.009^bB^	0.35 ± 0.005^aC^	0.47 ± 0.010^aA^	0.42 ± 0.020^bB^
12% oleogel	0.06 ± 0.003^cA^	0.07 ± 0.005^bB^	0.06 ± 0.007^bB^	0.32 ± 0.008^bA^	0.27 ± 0.008^cB^	0.24 ± 0.041^dC^

*Note:* Reported data are based on mean ± standard deviation (*n* = 3). Non‐common lowercase letters denote a significant difference at the 5% level of Duncan's test (*p* < 0.05) between the samples. Non‐common uppercase letters denote a significant difference at the 5% level of Duncan's test (*p* < 0.05) between the times.

#### 3.2.8 Sensory Analysis

Table 11 reveals significant differences in the sensory properties of the hamburger samples. As the replacement percentage increased, the color, taste, and overall acceptance scores of the samples decreased. Thus, substituting animal fat with black seed oil oleogel had an adverse impact on these parameters, resulting in reduced consumer acceptability of the hamburger samples. These findings are consistent with the results of the hamburger samples' color, which contained oleogel. However, in contrast, substituting animal fat with black seed oil oleogel had a positive impact on the texture and aroma of the hamburger samples, as the replacement percentage increased. These properties were found to be better than those of the control sample. The histological results of the hamburgers corroborate these findings.

**TABLE 11:** Results of sensory evaluation of hamburger samples after production.

Sensory characteristics					
Samples	Color	Taste	Texture	Aroma	Overall acceptance
Control	4.71 ± 0.03^a^	4.54 ± 0.04^a^	4.08 ± 0.04^d^	4.15 ± 0.00^d^	4.47 ± 0.03^a^
9% oleogel	4.33 ± 0.02^c^	3.78 ± 0.01^c^	4.36 ± 0.01^b^	4.43 ± 0.02^b^	3.90 ± 0.01^c^
6% oleogel	4.58 ± 0.02^b^	4.23 ± 0.02^b^	4.14 ± 0.02^c^	4.20 ± 0.00c	4.20 ± 0.00^b^
12% oleogel	4.11 ± 0.02^d^	3.76 ± 0.02^c^	4.72 ± 0.02^a^	4.61 ± 0.04^a^	3.62 ± 0.04^d^

*Note:* Reported data are based on mean ± standard deviation (*n* = 3). Non‐common lowercase letters denote a significant difference at the 5% level of Duncan's test (*p* < 0.05) between the samples.

## 4 Conclusions

This study aimed to investigate the combined impact of beeswax and adipic acid gelators on black seed oil oleogel, identify the optimal oleogel, and replace free oleogel at three levels in beef hamburgers to assess the effect of replacement and storage time on the hamburgers. The findings from the first part indicated that decreasing the percentage of adipic acid substitution in the samples increased the oleogels' oil storage capacity. However, increasing the adipic acid concentration heightened the samples' yellowness, brightness, and redness intensity, resulting in an overall blackish color for all samples. Increasing the adipic acid concentration led to a reduction in the samples' hardness, with the pure beeswax sample displaying the highest hardness. No synergistic effect was observed between the two gelators in oleogels. Based on the important parameters of texture and rheological characteristics for determining the sample's similarity to animal fat, sample B5 emerged as the optimal sample. In the second part of the study, replacing animal fat with the optimal black seed oil oleogel resulted in reduced oil absorption, cooking loss, and shrinkage of cooked hamburgers. However, over time, these parameters exhibited an increasing trend in the control sample and samples containing oleogel. The sensory characteristics of the cooked samples after production showed that color, taste, and overall acceptance were lower among consumers, but smell and texture were more acceptable compared to the control sample. These results suggest that the black seed oil oleogel can be used for production of hamburgers with favorable quality and nutritional characteristics.

## Author Approval and Responsibility Statement

All authors have read and approved the final version of the manuscript. Hadi Almasi had full access to all of the data in this study and takes complete responsibility for the integrity of the data and the accuracy of the data analysis.

## Author Contributions

**Hadi Almasi:** conceptualization, validation, supervision, resources, writing – review and editing, funding acquisition. **Fariba Zeynali:** visualization, project administration, supervision. **Shadieh Roon:** investigation, writing – original draft, methodology, formal analysis, software.

## Funding

The authors have nothing to report.

## Ethics Statement

The authors have nothing to report.

## Data Availability

The data that support the findings of this study are available on request from the corresponding author. The data are not publicly available due to privacy or ethical restrictions.
